# Social Disconnectedness, Perceived Loneliness, and Cognitive Functioning: The Role of Neighborhood Environment

**DOI:** 10.1093/geroni/igae009

**Published:** 2024-02-08

**Authors:** Fengyan Tang, Ke Li, Yi Wang, Yuyang Zhu, Yanping Jiang

**Affiliations:** School of Social Work, Univeristy of Pittsburgh. Pittsburgh, Pennsylvania, USA; School of Social Work, Univeristy of Pittsburgh. Pittsburgh, Pennsylvania, USA; School of Social Work, University of Iowa, Iowa City, Iowa, USA; School of Public Health, Rutgers, The State University of New Jersey, New Brunswick, New Jersey, USA; Department of Family Medicine and Community Health, Institute for Health, Health Care Policy and Aging Research, Rutgers, The State University of New Jersey, New Brunswick, New Jersey, USA

**Keywords:** Cognition, Immigration, Minority issues, Social isolation

## Abstract

**Background and Objectives:**

Social disconnectedness and loneliness pose significant challenges for older Chinese immigrants. Yet, it remains unclear whether they are associated with an increased risk of cognitive decline in this population. This study aimed to investigate the association of social disconnectedness and loneliness with cognitive functioning and examine the moderation role of neighborhood contexts.

**Research Design and Methods:**

This longitudinal analysis examined a sample of individuals aged 60 years and older from the Population Study of Chinese Elderly in Chicago (*N* = 2,044). Global cognition was assessed using the averaged *z*-scores of cognitive performance tests. Social disconnectedness was constructed using 5 indicators about structural aspects of social relationships. Loneliness was assessed with the R-UCLA loneliness scale. Neighborhood socioeconomic status (NSES) and neighborhood segregation index were constructed using 2010–2014 American Community Survey data at the census tract level. Individual perceptions about neighborhood environments were used to construct neighborhood cohesion index and neighborhood disorder index (NDI). Latent growth curve models with adjusted cluster robust standard errors were estimated.

**Results:**

More loneliness was associated with a higher level of initial cognitive functioning (*B* = 0.030, *p* < .01), but also with a faster decline rate over time (*B* = −0.007, *p* < .01) after adjusting for covariates. High NSES and less neighborhood segregation buffered the negative effects of loneliness on cognitive decline, respectively. High NDI amplified the positive relationship between loneliness and initial functioning, but accelerated the rate of cognitive decline associated with loneliness.

**Discussion and Implications:**

The study revealed that perceived loneliness, but not social disconnectedness, is a risk factor for cognitive decline among older Chinese immigrants. Living in a neighborhood with low socioeconomic status, more segregation, and high disorder elevated the detrimental effect of loneliness on long-term cognitive decline. Further research needs to investigate the complex interplay between social relationships, neighborhood environment, and cognition.

**Translational significance:** Given the rapid growth in older Chinese immigrant population and increasing prevalence of cognitive impairment, it is crucial to identify modifiable lifestyle and environmental factors that promote healthy cognitive aging. Our findings demonstrate that loneliness has a detrimental effect on cognitive decline, particularly among older Chinese immigrants residing in neighborhoods characterized by low socioeconomic status, high residential segregation, and high levels of physical and social disorder. By addressing loneliness and enhancing neighborhood conditions, we can potentially mitigate the negative impact of these factors on cognitive decline. This knowledge is vital for developing effective interventions and policies that improve cognitive health.

## Background and Objectives

Recent research has increasingly acknowledged the importance of social relationships in cognitive health among older adults. A number of studies assessed the associations of the objective and subjective aspects of social relationships, including social disconnectedness and perceived loneliness, with cognitive functioning (Evans, Martyr et al., 2019; Lara et al., 2019). The existing literature mostly documented the adverse effects of perceived loneliness, limited social support, small social networks, and low levels of social engagement on the increased risk of cognitive decline and impairment (Boss et al., 2015; Evans, Martyr et al., 2019; Harrington et al., 2023; Lara et al., 2019; Okamoto & Kobayashi, 2021). However, a few studies reported nonsignificant associations between various aspects of social relationships and cognitive measures (Boss et al., 2015; Harrington et al., 2023; Kuiper et al., 2016).

Social relationships are especially important for older immigrants during the process of adapting and aging in a new country. They often experience feelings of isolation and loneliness due to limited social networks, cultural differences, language barriers, discrimination, and changes in family dynamics (Lai et al., 2019; Li et al., 2018). These challenges are particularly pronounced for the rapidly growing population of older Chinese immigrants in the United States (Li et al., 2018). From 1980 to 2021, Chinese immigrant population increased by 543% from 370,000 to 2,380,000, representing the third largest origin group among U.S. immigrants, and over 20% of them were aged 60 and older (Rosenbloom & Batalova, 2023). About 26% of older adults in the U.S. Chinese community reported experiencing loneliness symptoms, in contrast to 16.9% in the general U.S. aging population during the period from 2000 to 2010 (Simon, Chang, Zhang et al., 2014).

Social disconnectedness and loneliness emerge as significant challenges faced by this population, potentially increasing the risk of cognitive decline (Li et al., 2018; Zhao et al., 2023). Furthermore, living environments play a crucial role in shaping cognitive health, and the impact of social relationships on cognitive functioning can vary by neighborhood contexts (Cassarino & Setti, 2015). As immigration in later life exacerbates social isolation and related stress, older immigrants may increasingly rely on their neighborhoods and social resources to alleviate stress levels, seek opportunities for social engagement, and develop supportive relationships (Wilmoth & Chen, 2003).

The objective of this study was to investigate the associations between social relationships and cognitive functioning among Chinese older adults residing in the Chicago area. Additionally, we assessed the moderating role of neighborhood contexts, including socioeconomic status, residential segregation, social cohesion, and neighborhood disorder in such associations. Our focus was on identifying potential risk factors for cognitive decline, examining both the objective, structural aspects of social disconnectedness, and the subjective experience of perceived loneliness. To address these objectives, we utilized panel data from the Population Study of Chinese Elderly (PINE), a population-based epidemiological study of U.S. Chinese older adults. This study aimed to answer the following research questions: (i) is there a significant association between social relationships and cognitive functioning among older Chinese immigrants, and (ii) does the neighborhood context moderate the association between social relationships and cognitive functioning?

## Two Aspects in Social Relationships: Social Disconnectedness and Loneliness

Social disconnectedness refers to the structural aspects of social relationships, marked by a lack of contact with others and limited social engagement (Cornwell & Waite, 2009; Okamoto & Kobayashi, 2021). This condition is manifested through situational factors such as living alone, being unmarried, a small size of social network, infrequent social interaction, or minimal involvement in social activities or groups (Cornwell & Waite, 2009; Okamoto & Kobayashi, 2021). By contrast, loneliness is a distressing perception of a discrepancy between one’s social desires and actual interactions, often indicated by subjective feelings of being socially isolated, and a perceived lack of social support (Cornwell & Waite, 2009; Russell et al., 2012).

According to a cognitive reserve perspective, maintaining a diverse range of social networks and actively engaging in social activities can offer mental stimulation, thereby fostering cognitive reserve, or the capacity to withstand cognitive decline caused by age-related brain pathology (Evans, Llewellyn et al., 2019; Stern, 2002, 2012). Evidence suggests that social disconnectedness poses a risk for cognitive impairment; yet the association between social disconnectedness and cognition remains inconclusive, with conflicting findings reported in the literature (Boss et al., 2015; Evans, Martyr et al., 2019; Harrington et al., 2023; Kuiper et al., 2016). Feelings of loneliness, on the other hand, are common among older adults and have profound consequences, including impaired cognitive performance, cognitive decline, and an increased risk of Alzheimer’s disease and related dementias (Hawkley & Cacioppo, 2010). Loneliness may contribute to mild cognitive impairment and dementia through various pathways, including biological, health behaviors, and psychosocial mechanisms (Evans, Llewellyn et al., 2019). Specifically, individuals who feel lonely are less likely to engage in intellectually stimulating activities, limiting the tolerance and recovery of neural networks from damages, and resulting in lower cognitive reserve and diminished cognitive functioning (Okamoto & Kobayashi, 2021; Stern, 2002, 2012).

Although previous research has generally indicated that various aspects of poor social relationships, including social networks, loneliness, social support, and social engagement, are related to an increased risk of cognitive decline and impairment, the overall conclusion remains tentative. Some studies reported that feelings of loneliness, rather than objectively being alone or social disconnectedness, are associated with a higher risk of dementia among nondemented community-dwelling older adults (Holwerda et al., 2014; Lara et al., 2019), whereas others found that objective measures of social disconnectedness, but not loneliness, predicted cognitive impairment (Beller & Wagner, 2018; Jang et al., 2021; Yu et al., 2023). The inconsistent findings in previous studies may be attributed to variations in the approaches to defining and assessing social isolation, especially the objective aspects, as well as differences in cognitive outcome measures (Evans et al., 2018). Moreover, a significant degree of heterogeneity across studies, including sample characteristics, research designs, covariates included, and statistical modeling approaches, may also contribute to inconsistency in findings (Harrington et al., 2023). The mixed findings in existing literature highlight the complexity in the nuanced relationships involved, and underscore the importance of identifying potential environmental factors that modify the associations of social disconnectedness and loneliness with cognitive functioning.

## The Role of Neighborhood Contexts

Compared with their U.S.-born peers, older immigrants are at a higher risk of experiencing cognitive decline and dementia, probably due to factors such as limited English proficiency and low acculturation (Franco & Choi, 2020). Also, older immigrants often reside in racially segregated and socioeconomically deprived neighborhoods, which offer limited opportunities for social engagement and inadequate access to health care, social services, and other essential resources (Tang et al., 2023). However, previous analyses revealed that residential segregation is not directly linked to cognitive functioning, whereas neighborhood socioeconomic advantage is associated with a slower rate of cognitive decline among older Chinese Americans, independent of significant individual-level factors including age and education (Guo et al., 2022; Tang et al., 2023). Although living in ethnically segregated neighborhood may initially offer some protection for newly arrived immigrants, prolonged exposure to residential segregation may contribute to cognitive decline (Tang et al., 2023).

In addition to the objective indicators of neighborhood environment—neighborhood socioeconomic status and residential segregation, subjective perceptions of neighborhoods may have a more direct impact on health (Lee & Waite, 2018). Perceived neighborhood cohesion may confer cognitive benefits through stimulating learning and facilitating opportunities for social activities, thereby influencing brain structure and function (Cassarino & Setti, 2015; Lee & Waite, 2018). By contrast, perceived neighborhood danger is associated with lower cognitive functioning, as living in a poorly built, stressful environment with high levels of decay and crime limits exposure to cognitive stimulation and potentially affects neurogenesis (Lee & Waite, 2018).

Neighborhood environments may play a moderating role in the associations of social disconnectedness and loneliness with cognitive functioning. Living in a socioeconomically advantaged and cohesive neighborhood holds the potential to mitigate the negative effects of social disconnectedness and loneliness on cognitive functioning. For instance, Stahl et al. (2017) suggest that older adults living alone had lower mental health risks when they perceived a high quality of neighborhood social cohesion, implying that social cohesion can serve as a protective factor against the detrimental health consequences of social isolation. Germane to this point, a recent study found cohesive neighborhoods ameliorated the negative effect of living alone on premature mortality among older Chinese immigrants (Jiang et al., 2023). In comparison, loneliness involves the feelings of unsafety or perceived social threat in the environment (Hawkley & Cacioppo, 2010). Immigrants, particularly those who migrated at an older age, may encounter greater challenges in navigating their environments and establishing social networks, which may lead to feelings of insecurity and withdrawal from social interactions (Lam, 2022). Yet, residing in favorable neighborhood conditions that facilitate social interactions, promote a sense of neighborhood identity, and enhance satisfaction with the local environment can make older immigrants less susceptible to loneliness and the associated negative health effects (Lam, 2022).

Despite the increasing number of studies on the topics, most research was conducted among non-Hispanic Whites, which may not be applicable to minority populations because of cultural and social variations (Boss et al., 2015). Given the fundamental values of collectivism and familism in Chinese culture, social relationships and neighborhood environment may hold particular significance and conditional effects on health maintenance among older Chinese immigrants. It is likely that the effects of social relationships on cognition are conditional on neighborhood contexts and underlying cultural mechanisms. We therefore anticipate that high levels of social disconnectedness and loneliness are associated with poorer cognitive functioning and a faster rate of cognitive decline. Further, we expect that the negative associations of social disconnectedness and loneliness with cognition are more pronounced among older Chinese immigrants residing in socioeconomically deprived, racially segregated, and disrupted neighborhoods.

## Research Design and Methods

### Sample

**Figure 1.**
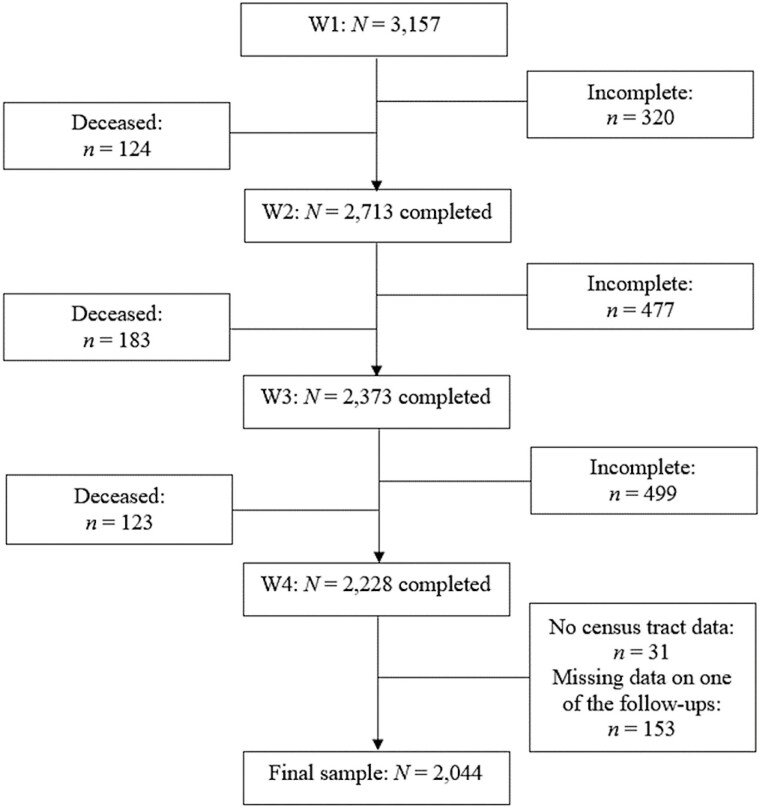
Flowchart of Population Study of Chinese Elderly in Chicago (PINE) study participants from Wave 1 to Wave 4. Participants who did not complete a previous wave were eligible and invited to participate in the subsequent wave.

Data were derived from the Population Study of Chinese Elderly (PINE), a community-engaged, population-based epidemiological study of U.S. Chinese older adults. A community-based participatory research approach was applied in recruiting participants from over 20 social service agencies in the Greater Chicago area (Simon, Chang, Rajan et al., 2014). The baseline started in 2011, with face-to-face interviews among 3,157 adults aged 60 and older, who self-identified as Chinese immigrants living in 170 census tracts in Chicago suburbs, north side/downtown, West, South, Southwest, and Chinatown. Three follow-up interviews were conducted during 2013–2015 (W2, *N* = 2,713; 86% of baseline), 2015–2017 (W3, *N* = 2,373; 75% of baseline), and 2017–2019 (W4: *N* = 2,228; 71% of baseline) among the original cohort (Figure 1). Respondents were interviewed in their preferred language: English (0.7%), Mandarin (22.5%), Cantonese (52.5%), or Taishanese (24.4%; Guo et al., 2022). The validated and widely used survey instruments in English were initially translated into Chinese, and subsequently, a back-translation was conducted to ensure content consistency between the Chinese version and the original English version (Chang & Dong, 2014).

In this study, we linked the geocoded data of the participants’ residential addresses to neighborhood-level variables, specifically neighborhood socioeconomic status (NSES) and neighborhood segregation index (NSI). These two variables were calculated based on the 2010–2014 American Community Survey (ACS) 5-year estimates at the census tract level.

A total of 2,075 participants completed four waves of interviews, of which 31 participants were excluded due to missing information on census tracts. The final analysis, thus, included 2,044 respondents. Sensitivity analysis was conducted using baseline data. Compared with respondents included in the analysis, those who were excluded had poorer cognitive and physical health, experiencing more disconnectedness and loneliness, and reporting less social support and social engagement. They were older, less educated, with more income, longer residence years in the United States, and higher acculturation, living in less-cohesive, low-NSES, or more segregated toward Chinese-speaking neighborhoods (Supplementary Table 1).

### Measures

All individual-level predictors and covariates were measured at baseline. Cognitive functioning was assessed across four waves.

#### Global cognition

A battery of cognitive tests was administrated to evaluate cognitive functioning, including the East Boston Story Tests—Immediate Recall, Delayed Recall, the Digit Span Backwards assessment drawn from the Wechsler Memory Scale—Revised, the oral version of the Symbol Digit Modalities Test, and the Chinese version of the Mini-Mental State Examination (Guo et al., 2022; Li et al., 2017). Using the population estimates of the mean and standard deviation (*SD*), we first converted raw scores of these tests to *z*-scores to construct cognitive domains of episodic memory, working memory, perceptual speed, and mental status. Then we averaged these *z*-scores to create a composite *global cognition* to minimize floor and ceiling artifacts and other measurement errors (Li et al., 2017). Principal component analysis indicated that these five tests had loadings between 0.72 and 0.87 on the first component, accounting for 67% of the variance (Li et al., 2017). Higher scores indicate better overall cognitive functioning (*α* = 0.86).

#### Social disconnectedness

Following Cornwell and Waite (2009), we first selected 11 indicators, including social network size, contact frequency, structural components (i.e., proportions of kin, female, or coresidence), emotional closeness, household size, living arrangement, and marital status. We then removed six items with low item-rest correlation to maximize internal consistency reliability. Five items were retained to create the social disconnectedness index, including social network size (number of social network ties with whom one can discuss important things), social network range (diversity of social network ties), household size (number of people living in the same household), living alone or not, and having spouse/partner or not. The average standardized score of these indicators was used, with higher scores indicating greater social disconnectedness (*α* = 0.70).

#### Perceived loneliness

We calculated a summary score using three items from the Revised—University of California Los Angeles Loneliness (R-UCLA) Scale (Hughes et al., 2004). Participants were asked about their feelings of lacking companionship, being left out, and being isolated from others. Responses were given on a 3-point scale, including hardly ever, sometimes, and often. A higher score on the summary scale indicated greater loneliness (*α* = 0.75).

#### Neighborhood characteristics

We used two objective measures, that is, NSES and NSI, and two subjective evaluations, that is, neighborhood cohesion index (NCI) and neighborhood disorder index (NDI). Following previous literature (Howard et al., 2016; Stulberg et al., 2021), NSES was constructed using six variables obtained from the ACS (2010–2014) estimates at the census tract level. These variables included the logarithm of median household income, the logarithm of median value of owner-occupied housing units, the proportion of households receiving interest, dividend, or net rental income, the proportion of adults aged 25 and above with a high school diploma, the proportion of adults aged 25 and above with a college degree, and the proportion of people employed in executive, managerial, or professional occupations. Each variable was standardized, and the sum of standardized scores was calculated, with high values indicating favorable NSES.

In accordance with previous studies (Feldman et al., 2015; Krieger et al., 2016; Ward et al., 2018), we utilized language use data from the ACS (2010–2014) estimates to construct the NSI. The NSI was calculated as the ratio of the difference between the number of English-only speakers and the number of Chinese speakers to the total population for whom language preference is known in a given census tract (Ward et al., 2018). The index offers advantages over other segregation measures used in previous literature (e.g., proportion of a racial/ethnic population), as it can capture neighborhoods with either 100% Chinese speakers or 100% English-only speakers, represented by values of −1 and 1, respectively. Consequently, the index effectively reflects spatial social polarization or the spatial distribution of racial/ethnic groups (Feldman et al., 2015).

The NCI consisted of six items adapted from the Chicago Neighborhood and Disability Study (CNDS), which assessed individual integration and the overall cohesiveness in the neighborhood (Cagney et al., 2009). These items included familiarity with neighbors, social interactions, and mutual assistance among neighbors. Given that the items utilized different response scales, a composite score was constructed by averaging the standardized score of each item, with higher scores indicating a greater level of cohesion in the neighborhood (*α* = 0.86).

The NDI comprised eight items derived from the CNDS, which assessed physical and social disorder in the neighborhood (Cagney et al., 2009). The items included the presence of trash and litter, strangers, speeding cars, vandalism, unsafe conditions for walking, and poorly maintained sidewalks or lightings. Respondents indicated the frequency of their observations on a scale ranging from 0 (never) to 3 (often). A sum score was calculated by adding up the responses, and higher scores indicated more disorder perceived in the neighborhood (*α* = 0.81).

#### Covariates

Covariates included social support, social engagement, sociodemographic, immigration-related variables, and health status, which are significant correlates of cognitive functioning among immigrants (Guo et al., 2022). Social support was assessed with six items rating the extent to which one opened up to and relied on their spouse, family members, and friends. A summary score was used, and higher scores indicated higher levels of perceived social support (*α* = 0.73). Social engagement was measured with 16 items about frequency of engagement in various activities, such as reading, visiting friends, attending concerts. A higher score indicated more frequent social engagement (*α* = 0.75).

Sociodemographics included age (years), female (1 = yes, 0 = no), education (years), personal income (range: 1 = less than $5,000 to 10 = $45,000 or more), years living in the United States (range: 0.1–90). Acculturation was calculated using a 12-item multidimensional scale of individual preferred language use in different settings and preferred ethnicity they interacted with. Responses were given on a 5-point scale, from 1 (only Chinese) to 5 (only English). The sum score ranged from 12 to 60, with higher scores indicating a higher level of acculturation (*α* = 0.92). Instrumental daily living limitations (instrumental activities of daily living [IADL]) were assessed with a scale measuring the level of difficulty in performing various IADLs (*α* = 0.90). Depressive symptoms were evaluated using the Patient Health Questionnaire-9 (*α* = 0.82).

### Statistical Analysis

We used latent growth curve modeling (LGCM) to examine the trajectory of global cognition with two latent factors: the intercept, which represents the initial level of cognitive functioning, and the slope, which indicates the rate of change over time. The analysis was conducted in several steps. We first estimated unconditional linear and quadratic models, and four waves of data collection were scaled at 0, 2, 4, and 6, respectively. Models were considered to have a good fit to data if the comparative fit index (CFI) and the Tucker–Lewis index (TLI) ≥ 0.95, and the root-mean-squared error of approximation (RMSEA) ≤ 0.06 (Hu & Bentler, 1999). The unconditional linear model provided good fit to the data (χ^2^(8) = 91.34, CFI = 0.98, TLI = 0.98, RMSEA = 0.07). However, the unconditional quadratic model did not fit the data, as the latent variables were not statistically distinguishable with a negative covariance matrix, and the variance of the quadratic slope did not reach statistical significance. Thus, we selected the linear model as the final unconditional model, and results showed that both initial cognition level (*M* = 0.05, *p* < .01) and decline rate over time (*M* = −0.07, *p* < .001) varied across individuals.

We then estimated a conditional model by introducing social disconnectedness and loneliness to the unconditional linear model (Model 1). In Model 2, we added social support and social engagement to control for their additional contributions. Afterward, we sequentially incorporated all individual-level covariates and a single neighborhood variable into Models 3–6, respectively. This approach was adopted given the notable correlations among neighborhood variables (Supplementary Table 2). Lastly, we created eight interaction terms of social disconnectedness and loneliness with NSES, NSI, NCI, and NDI, and tested them in eight separate models after controlling for covariates. All variables were centralized when creating interaction terms to avoid multicollinearity. The sequential analyses allowed us to examine the extent to which the main effects of social disconnectedness and loneliness would change after controlling for covariates and whether these effects varied by neighborhood contexts. We further conducted sensitivity analyses to account for potential effects of time-varying covariates (i.e., social support, social engagement, depressive symptoms, and IADL).

Missing information was observed across the study variables, with the most missing cases in IADL (*n* = 57) and NCI (*n* = 50). Missing data were handled using the full-information maximum-likelihood estimation method, which provides robust estimates with all available data. All analyses were conducted with Mplus 8.8 (Muthén & Muthén, 2012), using the cluster-adjusted standard errors to account for neighborhood clustering (McNeish et al., 2017).

## Results

The sample characteristics are presented in Table 1. The mean age of the sample was 72 (range 59–97 years) at baseline, and 60% were female. The average standardized cognition scores at Waves 1–4 were 0.02, −0.03, −0.18, and −0.37, respectively, indicating a declining trend over time. The study participants resided in the neighborhoods with a mean NSI score of 0.04 (*SD* = 0.52, range −0.66 to 0.94) and a mean NSES score of −0.40 (*SD* = 2.78, range −6.92 to 11.78).

**Table 1.** Descriptive Statistics of the Population Study of Chinese Elderly in Chicago (PINE) Participants and Neighborhoods (*N* = 2,044)

Variable	*M*	*SD*	Range
Time 1 cognition	0.02	0.77	−3.00 to 1.77
Time 2 cognition	−0.03	0.80	−3.23 to 2.16
Time 3 cognition	−0.18	0.83	−3.09 to 1.52
Time 4 cognition	−0.37	0.95	−3.12 to 1.61
Social disconnectedness	0.002	0.68	−1.48 to 1.85
Loneliness	0.52	1.13	0–6
Social engagement	21.41	9.07	0–57
Social support	12.98	3.01	6–18
Age	71.52	7.54	59.39–96.96
Female	0.60	0.49	0–1
Income	1.91	1.07	1–10
Education	8.86	5.03	0–26
Years living in the United States	18.41	11.62	0.1–90
Acculturation	15.04	4.33	12–51
Instrumental Activities of Daily Living	2.79	4.92	0–36
Depressive symptoms	2.56	4.00	0–27
Neighborhood disorder index	4.07	4.12	0–22
Neighborhood cohesion index	0.07	0.79	−1.07 to 2.83
Neighborhood segregation index	0.04	0.52	−0.66 to 0.94
Neighborhood socioeconomic status	−0.40	2.78	−6.92 to 11.78

*Notes*: PINE = Population Study of Chinese Elderly in Chicago; *SD* = standard deviation.

**Figure 2.**
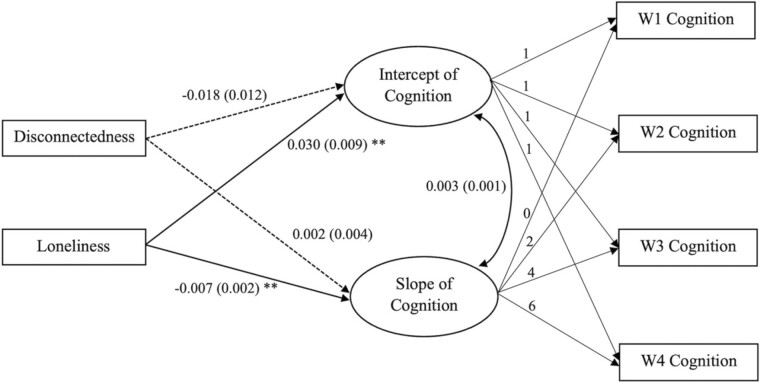
Latent growth curve modeling of main effects of social disconnectedness and loneliness on cognition intercept and slope. Estimates (standard error) were displayed. Covariates were included in the model but not displayed for simplicity. Dash lines represent statistically nonsignificant (*p* > .05). **p < .01.

Table 2 presents results of LGCM models. Fit indices indicated good model fit in all models. Model 1 showed that social disconnectedness was negatively associated with initial cognition level (*B* = −0.199, *p* < .001) and the slope (*B* = −0.009, *p* < .05), indicating a higher level of social disconnectedness was associated with a lower initial level of cognitive functioning and a faster decline rate over time. More loneliness was significantly associated with a faster decline in cognition (*B* = −0.005, *p* < .05), but not associated with the initial level. In Model 2, after controlling for social support and social engagement, social disconnectedness remained significantly negatively associated with cognition, whereas loneliness turned out to be positively associated with the initial cognition level (*B* = 0.039, *p* < .01), and remained the same negative relationship with the slope as in Model 1. In Models 3–6, social disconnectedness lost its significant associations, but loneliness remained significantly associated with both the intercept and the slope of cognitive trajectory, independent of covariates considered in the model (full results of Models 4–6 in Supplementary Table 3). Figure 2 illustrates the main effects of social disconnectedness and loneliness after controlling for all individual covariates and NSES. Regarding neighborhood characteristics, NDI was significantly related to the intercept of cognitive trajectory. Individuals perceiving more neighborhood disorder had better initial functioning (*B* = 0.005, *p* < .05). NSES was significantly associated with the slope, indicating that individuals living in a higher NSES neighborhood exhibited a slower decline in cognitive functioning over time (*B* = 0.002, *p* < .05). NSI and NCI were not associated with either the intercept or the slope of cognitive trajectory.

**Table 2.** Latent Growth Curve Modeling Analysis of Social Relationships, Neighborhood Characteristics, and Global Cognition

Predictors	Model 1				Model 2				Models 3–6			
	Intercept		Slope		Intercept		Slope		Intercept		Slope	
	Estimate	*SE*	Estimate	*SE*	Estimate	*SE*	Estimate	*SE*	Estimate	*SE*	Estimate	*SE*
Disconnectedness	−0.199***	0.025	−0.009*	0.004	−0.153***	0.022	-0.009*	0.004	−0.018	0.012	0.002	0.004
Loneliness	0.008	0.015	−0.005*	0.002	0.039**	0.013	-0.005*	0.002	0.030**	0.009	−0.007**	0.002
Social engagement					0.042***	0.002	0.000	0.000	0.017***	0.002	0.000	0.000
Social support					0.024***	0.005	0.000	0.001	0.013***	0.003	−0.001	0.001
Age									−0.019***	0.002	−0.003***	0.000
Female									−0.038	0.023	0.012**	0.004
Income									0.029**	0.011	−0.001	0.002
Education									0.061***	0.003	0.002**	0.001
Acculturation									0.007	0.004	0.000	0.000
Years in United States									0.002	0.001	0.000	0.000
IADLs									−0.032***	0.004	−0.001	0.001
Depression									−0.006	0.004	0.001	0.001
NSES									−0.004	0.006	0.002*	0.001
NCI^4^									0.011	0.015	−0.003	0.003
NSI^5^									0.025	0.033	0.002	0.004
NDI^6^									0.005*	0.002	0.000	0.001
Model fit												
χ²(*df*)	112.67(12)***				131.54(16)***				174.00(34)***			
CFI	0.978				0.979				0.992			
TLI	0.974				0.971				0.987			
RMSEA	0.064				0.059				0.045			

*Notes*: CFI = comparative fit index; IADLs = instrumental activities of daily living; NCI = neighborhood cohesion; NDI = neighborhood disorder; NSES = neighborhood socioeconomic status; NSI = neighborhood segregation; RMSEA = root-mean-square error of approximation; *SE* = standard error; TLI = Tucker–Lewis index. Model estimate and standard error were reported. Model 3 included NSES and individual-level covariates. NDI, NCI, and NSI were estimated in separate Models 4–6 with individual-level variables.

*p < .05. **p < .01. ***p < .001.

**Figure 3.**
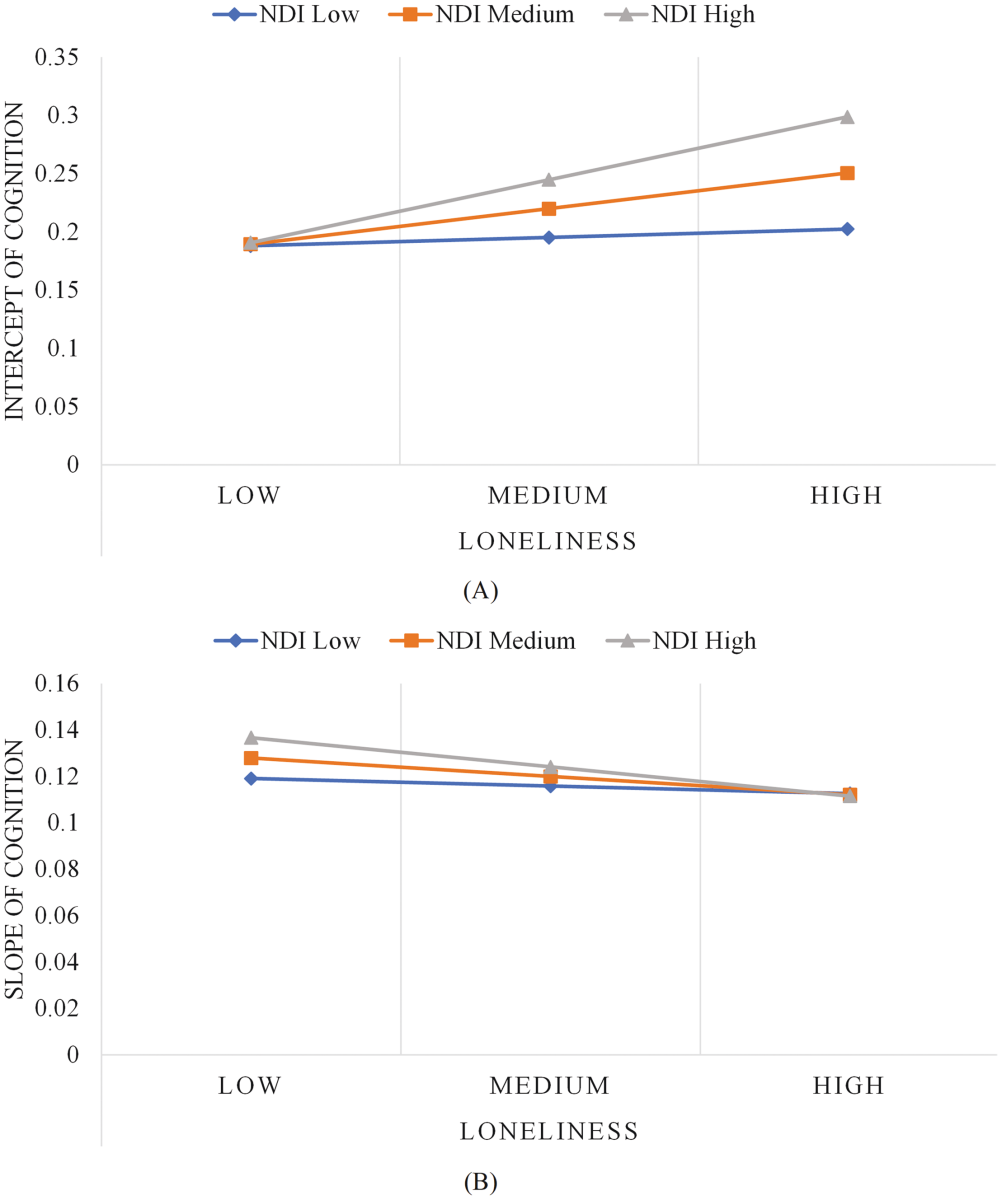
Moderation effect of NDI on (A) initial cognitive status and (B) cognitive decline. NDI = neighborhood disorder. NDI: low = 0, medium = 4.1, high = 8.2. Loneliness: low = 0, medium = 0.5, high = 1.6.

Table 3 presents the interactive effects of social disconnectedness and loneliness with NCI, NDI, NSES, and NSI, which were tested in eight separate models after controlling for covariates. Results indicated that only NDI modified the relationship between loneliness and initial cognitive status (*B* = 0.005, *p* < .01). Specifically, residing in a high disorder neighborhood amplified the positive relationship between loneliness and initial cognitive status. Further, NDI, NSI, and NSES modified the relationship of social disconnectedness and loneliness with cognitive decline, but in opposite directions. High NSES (*B* = −0.003, *p* < .05) and high NSI (or segregation toward English-only speakers; *B* = −0.018, *p* < .001) exacerbated the potentially negative effects of social disconnectedness on cognitive decline, but buffered against the loneliness effect on cognitive decline, respectively (NSES: *B* = 0.002, *p* < .05; NSI: *B* = 0.007, *p* < .05). Although high NDI hastened loneliness-related cognitive decline (*B* = −0.001, *p* < .05), it might slow cognitive decline associated with social disconnectedness (*B* = 0.002, *p* < .05). Figure 3 depicts the moderation effects of NDI, with low, medium, and high values designated as 1 *SD* below the mean, at the mean, and 1 *SD* above the mean, respectively (figures for other significant moderation effects available upon request). Sensitivity analyses confirmed the significant impact of loneliness on cognitive decline; however, the main effects of NDI and NSES were no longer statistically significant (Supplementary Table 4). Moreover, although the strength of the moderation effects of neighborhood contexts mentioned earlier was slightly reduced, most of these moderation effects remained statistically significant (Supplementary Table 5).

**Table 3.** Moderation Effects of Social Relationships and Neighborhood Characteristics on Global Cognition

Interactive terms	Intercept	Slope
	Estimate (*SE*)	Estimate (*SE*)
Disconnect*NCI	−0.004 (0.026)	0.004 (0.005)
Loneliness*NCI	0.010 (0.015)	−0.002 (0.002)
Disconnect*NDI	−0.002 (0.003)	0.002 (0.001)*
Loneliness*NDI	0.005 (0.002)**	−0.001 (0.000)*
Disconnect*NSES	−0.002 (0.006)	−0.003 (0.001)*
Loneliness*NSES	−0.004 (0.003)	0.002 (0.001)*
Disconnect*NSI	−0.009 (0.019)	−0.018 (0.005)***
Loneliness*NSI	−0.006 (0.013)	0.007 (0.003)*

*Notes*: NCI = neighborhood cohesion; NDI = neighborhood disorder; NSES = neigborhood socioeconomic status; NSI = neighborhood segregation; *SE* = standard error. Model estimate and standard error were reported. Eight separate models were estimated with each interaction term after controlling for individual-level variables.

*p < .05. **p < .01. ***p < .001.

## Discussion

In this study, we investigated the associations between social relationships and cognitive functioning among older Chinese immigrants, and assessed the moderating effects of neighborhood characteristics on the associations. The study findings revealed that perceived loneliness, but not social disconnectedness, was a risk factor for cognitive decline. Importantly, the impact of loneliness on cognitive decline was found to be contingent on the socioeconomic status, residential segregation, and disorder level in the neighborhood. Living in a neighborhood with higher NSES or with more English-only speaking residents could potentially mitigate the negative effects of loneliness on cognitive decline. Conversely, living in a neighborhood characterized by poor infrastructure, insecurity, and deprivation may exacerbate the detrimental effect of loneliness on cognitive decline over time. These results highlight the complex interplay between loneliness, neighborhood environment, and cognitive functioning in older Chinese immigrants.

The findings suggest the intricate nature of the associations between social relationships and cognitive functioning. Although bivariate correlations among the observed variables were largely consistent with previous research, the significant effect of social disconnectedness diminished and became nonsignificant after adjusting for individual- and neighborhood-level covariates in the LGCM models, implying that some potential factors mediate or confound the associations between social disconnectedness and cognition. Such findings implied that the association between social relationships and cognition might be overestimated in previous studies (Evans, Martyr et al., 2019; Kuiper et al., 2016). Moreover, as discussed in Beller and Wagner (2018), most of the existing research has been conducted in individualistic Western countries, where there tends to be a higher prevalence of objective social disconnection when compared to collectivistic, non-Western countries. Though social disconnectedness may pose great health risks among older adults in Western countries, it may not serve as an independent risk factor contributing to adverse health outcomes in Chinese or other Asian older adults (Chou et al., 2006; Gu et al., 2019). Indeed, Chinese older adults living alone may perceive their overall health more positively than those living with others (Tang, Li, & Jang, 2023), suggesting that the health effects of living alone as a component of social disconnectedness may vary across different cultural contexts. Cultural norms and values surrounding social connectedness may influence individuals’ perceptions of health and well-being. Particularly, filial piety as a core family norm in Chinese culture has substantial impacts on psychological well-being (Guo et al., 2020). Chinese older adults prefer to rely on their children for social support, making social connectedness with friends or other family members less consequential (Kim & Silverstein, 2021). It is crucial to take into account cultural diversity when interpreting findings regarding social relationships and health outcomes in later life.

Consistent with prior research (Boss et al., 2015; Ellwardt et al., 2013; Harrington et al., 2023), our study confirmed that loneliness, or the perceived deficit in social relationships, is a crucial risk factor for cognitive decline, after controlling for sociodemographic, physical and mental health, social activity, and neighborhood contexts. The controversy of the cross-sectional association between loneliness and cognition documented in the study may be attributed to the presence of strong social support and active social engagement that mitigate the immediate cognitive effects of loneliness, particularly, in our study, the sample with a low level of loneliness (*M* = 0.53), whereas previous research indicated that only severe loneliness is consistently associated with impaired cognitive functioning (Cacioppo & Hawkley, 2009; Ellwardt et al., 2013). For older immigrants who face numerous challenges, increasing social support and activity engagement may play a prominent role in promoting a proactive lifestyle and reducing feelings of loneliness. As loneliness remains a risk factor for cognitive decline, interventions aimed at reducing loneliness and promoting social support are critical for maintaining cognitive health in older Chinese immigrants.

Another key finding in this study reveals the influence of neighborhood contexts on the relationship between loneliness and cognitive decline. Our results demonstrate that certain neighborhood characteristics, such as socioeconomic resources, reduced segregation, and decreased disorder, can serve as protective factors that alleviate the long-term adverse impacts of loneliness on cognitive decline. Specifically, high NSES has both significant independent and interactive effects, suggesting that residing in a high NSES neighborhood can buffer the negative effects of loneliness on cognitive decline. Although NSI did not directly affect cognition, it may exhibit similar moderating effects on cognitive decline as NSES, implying that living in socially integrated and economically advantaged neighborhoods could decelerate cognitive decline over time. Although the main effect of neighborhood disorder was not significant, it is important to note that prolonged exposure to physically and psychosocially challenging environments may expedite the detrimental consequences of loneliness on cognitive decline. These findings underscore the important role of neighborhood contexts in shaping the relationship between loneliness and cognitive decline.

Unexpectedly, our study uncovered a positive association between loneliness and initial cognitive functioning, accentuated by the influence of neighborhood disorder. It is possible that individuals with high cognitive functioning tend to report feelings of loneliness and perceive their neighborhood as disorderly. These individuals might include recently arrived immigrants with higher education levels and cognitive abilities in comparison to their U.S.-born counterparts. Their loneliness may, in part, stem from limited social connections, cultural identity conflicts, and intergenerational discord (Albert, 2021). They might choose to live in an ethnically concentrated neighborhood, often marked by socioeconomic deprivation and disruptive or unsafe conditions, yet fostering a sense of community and identification among co-ethnic residents (Lam, 2022). Additionally, it is important to note that loneliness is not a persistent state, and older adults can transition in and out of loneliness status over time (Hawkley & Kocherginsky, 2017).

Although a significant main effect of social disconnectedness was absent, our study revealed intriguing moderation effects of certain neighborhood characteristics, albeit in contrast to the patterns observed in the loneliness–cognition relationship. Specifically, neighborhood disadvantages, such as lower socioeconomic status, increased segregation toward Chinese-speaking residents, and heightened disorder, appeared to potentially mitigate cognitive decline associated with social disconnectedness. The findings imply that individual experiences in such neighborhoods, such as fitting in the environment, experiencing low levels of acculturative stress, fewer conflicts, or reduced discrimination, may be relevant factors contributing to these protective effects (Albert, 2021). Nevertheless, such findings should be interpreted with caution.

Our study aligns with prior research that underscores the role of the neighborhood environment in shaping cognitive health in older adults (Besser et al., 2017). However, we did not find the significant main effect of residential segregation or social cohesion on cognition. The health outcomes associated with residential segregation are mixed and vary by race/ethnicity along with the underlying mechanisms driving segregation (Ward et al., 2018). Although living in a segregated neighborhood may offer easy access to cultural/ethnic resources and within-group support, the residential conditions of older Chinese immigrants are often characterized by a poor built environment, low-quality education, unfavorable health behaviors, and psychosocial stress, which may negatively contribute to cognitive aging (Guo et al., 2022).

There are limitations in our analysis. Although the PINE study provides valuable insights into the Chinese aging population in the Greater Chicago area, caution is warranted when attempting to generalize our findings to other aging populations or older Chinese immigrants in different regions. Our study sample exhibited a bias toward a relatively healthy and less socially isolated population, which may lead to an underestimation of the associations between social relationships and cognition. Additionally, about 23% of study participants resided in the Chinatown census tract, potentially introducing bias in the estimation of neighborhood contexts and complicating the interactive effect with social relationships on cognition. Furthermore, the limited set of indicators used for measuring social disconnectedness omitted critical elements, such as contact frequency, due to their minimal contribution to internal reliability in preliminary analysis. Although we controlled for social support and social engagement, the current study did not fully capture the dynamic and interactive nature of these aspects. For instance, individuals may experience loneliness even when engaging in solitary activities that lack social interaction but may still provide cognitive stimulation. Lastly, upon adjusting for some covariates that might be time varying, the main effects of neighborhood contexts seemed to diminish. These findings suggest potential implications arising from the lack of control over between-person variances in these time-varying covariates. Future studies should explore the interrelated, dynamic nature of these factors concerning cognitive decline.

Our study has several strengths. We leveraged longitudinal data and geocoded information from a population-based sample, which is representative of older Chinese immigrants residing in Chicago. The utilization of the PINE study, which benefits from a community-based participatory research approach, allowed us to comprehensively examine the intricate interplay between individual and environmental factors in cognitive aging. Moreover, our study benefited from a culturally and linguistically sensitive research infrastructure. We employed trained bilingual/bicultural interviewers and validated instruments for data collection, ensuring that the study was culturally relevant in its assessment. To our knowledge, no existing studies have comprehensively evaluated both the objective and subjective aspects of social relationships and neighborhood contexts in this understudied minority population.

The study findings underscore the detrimental effect of loneliness on cognitive decline, while also highlighting the protective effects of neighborhood socioeconomic advantages, reduced segregation, and safety. To mitigate cognitive risks associated with loneliness, it is imperative to address modifiable risk factors. This includes promoting involvement in community organizations, support groups, or volunteer opportunities, and strengthening social support networks with family, friends, and community. Further, concerted efforts should be directed toward addressing neighborhood issues, including physical disorder, public safety, residential segregation, and economic disparities. Loneliness can pose particular challenges for older Chinese immigrants, given language barriers, cultural differences, and limited social networks. In particular, working with newly arrived immigrants requires a culturally sensitive and holistic approach that considers their social, emotional, and environmental needs. Building trust and rapport within the community is essential to reducing social isolation and facilitating integration into their new environment. It is also important to provide cultural and language support, assist with family reunification and intergenerational connections, and make public transportation and social services more accessible.

## Conclusion

The study is one of the pioneering attempts to investigate the complex relationships among social disconnectedness, loneliness, neighborhood contexts, and cognitive functioning in older Chinese immigrants. The findings shed light on the potential adverse effects of loneliness on cognitive decline. Moreover, the study implies that older Chinese immigrants may be less susceptible to cognitive impairment stemming from socially isolated circumstances when provided with opportunities to broaden their experiences through social integration and engaging in socially and cognitively stimulating activities.

Recognizing the significance of social relationships and neighborhood contexts in cognitive health lays a crucial foundation for the development and implementation of strategies and programs aimed at promoting social integration, alleviating loneliness, and cultivating supportive neighborhood environments for older Chinese immigrants and other minority aging populations. These initiatives hold the promise of preserving cognitive health and enhancing overall well-being among older adults.

## Supplementary Material

igae009_suppl_Supplementary_Tables_S1-S5

## Data Availability

The data, analytic methods, or materials will be available to other researchers for replication purposes upon request to the senior author. The study was not preregistered.
