# Supplementary material for: Social Disconnectedness, Perceived Loneliness, and Cognitive Functioning: The Role of Neighborhood Environment
Source: Innov Aging. 2024 Feb 8;8(2):igae009. doi: 10.1093/geroni/igae009 (PMC10946307; doi:10.1093/geroni/igae009)
Supplement: igae009_suppl_Supplementary_Tables_S1-S5 [file igae009_suppl_supplementary_tables_s1-s5.docx]

*Innovation in Aging* Supplementary Material: Fengyan Tang, Ke Li, Yi Wang, Yuyang Zhu, & Yanping Jiang. Social Disconnectedness, Perceived Loneliness, and Cognitive Functioning: The Role of Neighborhood Environment.

**Supplemental Table 1.** *Comparison Between the Dropped and the Retained in the Study at Baseline Characteristics (N=3,157)*

| **Variable** | **Mean (SD)** | | **t-test/chi-square** |
| --- | --- | --- | --- |
|  | **Dropped**  **(n= 1113)** | **Retained**  **(n=2044)** |  |
| Global Cognition | -0.18 (0.90) | 0.04 (0.76) | -6.93 (1894.5)*** |
| Social disconnectedness | 0.08 (0.69) | -0.04 (0.67) | 4.83 (3155)*** |
| Loneliness | 0.71 (1.35) | 0.52 (1.13) | 3.97 (1921.1)*** |
| Social engagement | 19.66 (9.29) | 21.41 (9.07) | -5.14(3138)*** |
| Social support | 12.27 (3.14) | 12.98 (3.01) | -6.13 (2193.3)*** |
| Age | 75.19 (9.09) | 71.52 (7.54) | 11.46 (1951.8)*** |
| Female | 55% | 60% | 6.51 (1)* |
| Income | 2.02 (1.25) | 1.91 (1.07) | 2.66(3119)* |
| Education | 8.46 (5.10) | 8.86 (5.03) | -2.13 (3136)* |
| Years living in the US | 22.97 (15.23) | 18.41 (11.62) | 8.67 (1815.2)*** |
| Acculturation | 15.65 (6.30) | 15.04 (4.33) | 2.88 (1693.5)** |
| Instrumental activities of daily living | 5.53 (8.22) | 2.79 (4.92) | 10.01 (1497.7)*** |
| Depressive symptoms | 2.82 (4.35) | 2.56 (4.00) | 1.68 (2097.5) |
| Neighborhood disorder index | 3.99(4.05) | 4.07 (4.12) | -0.52 (3116) |
| Neighborhood cohesion index | -0.13 (0.71) | 0.07 (0.79) | -6.94 (2384.9)*** |
| Neighborhood segregation index | -0.03 (0.53) | 0.04 (0.52) | -3.10 (2073.7)** |
| Neighborhood socioeconomic status | -0.61 (2.68) | -0.40 (2.78) | -2.05(2196.1)* |

**Supplemental Table 2.** *Correlation among Study Variables at Baseline*

|  | Variable | 1 | 2 | 3 | 4 | 5 | 6 | 7 | 8 | 9 | 10 | 11 | 12 | 13 | 14 | 15 | 16 |
| --- | --- | --- | --- | --- | --- | --- | --- | --- | --- | --- | --- | --- | --- | --- | --- | --- | --- |
| 1 | Cognition | _ |  |  |  |  |  |  |  |  |  |  |  |  |  |  |  |
| 2 | Social disconnected | -0.17  *** | _ |  |  |  |  |  |  |  |  |  |  |  |  |  |  |
| 3 | Loneliness | -0.01 | 0.20  *** | _ |  |  |  |  |  |  |  |  |  |  |  |  |  |
| 4 | Social support | 0.22  *** | -0.38  *** | -0.21  *** | _ |  |  |  |  |  |  |  |  |  |  |  |  |
| 5 | Social engagement | 0.51  *** | -0.03 | -0.07  *** | 0.19  *** | _ |  |  |  |  |  |  |  |  |  |  |  |
| 6 | Age | -0.28  *** | 0.32  *** | 0.05  * | -0.18  *** | -0.03 | _ |  |  |  |  |  |  |  |  |  |  |
| 7 | Female | -0.16  *** | 0.19  *** | 0.03 | -0.08  *** | -0.16  *** | -0.00 | _ |  |  |  |  |  |  |  |  |  |
| 8 | Education | 0.60  *** | -0.11  *** | 0.04 | 0.17  *** | 0.55  *** | -0.06  ** | -0.19  *** | _ |  |  |  |  |  |  |  |  |
| 9 | Income | 0.14  *** | 0.07  ** | -0.01 | 0.00 | 0.10  *** | -0.04 | 0.02 | 0.13  *** | _ |  |  |  |  |  |  |  |
| 10 | Acculturation | 0.31  *** | 0.03 | 0.01 | -0.00 | 0.34  *** | -0.01 | -0.03 | 0.39  *** | 0.32  *** | _ |  |  |  |  |  |  |
| 11 | IADLs | -0.40  *** | 0.20  *** | 0.15  *** | -0.16  *** | -0.26  *** | 0.39  *** | 0.11  *** | -0.16  *** | -0.05 | -0.10  *** | _ |  |  |  |  |  |
| 12 | Depression | -0.15  *** | 0.10  *** | 0.47  *** | -0.21  *** | -0.20  *** | 0.06  ** | 0.11  *** | -0.02 | -0.08  *** | -0.02 | 0.32  *** | _ |  |  |  |  |
| 13 | NDI | -0.04 | 0.14  *** | 0.07  *** | -0.06  ** | -0.03 | -0.10  *** | -0.00 | -0.09  *** | 0.05  * | -0.08  *** | 0.03 | 0.09  *** | _ |  |  |  |
| 14 | NCI | 0.12  *** | 0.11  *** | -0.05  ** | 0.15  *** | 0.29  *** | 0.14  *** | 0.07  ** | 0.15  *** | 0.03 | 0.08  *** | -0.00 | -0.08  *** | 0.03 | _ |  |  |
| 15 | NSES | 0.22  *** | -0.06  * | 0.04 | 0.08  *** | 0.24  *** | 0.11  *** | -0.01 | 0.38  *** | 0.01 | 0.21  *** | -0.01 | 0.06  ** | -0.24  *** | 0.11  *** | _ |  |
| 16 | NSI | 0.25  *** | -0.08  *** | 0.02 | 0.08  *** | 0.25  *** | -0.02 | -0.04 | 0.37  *** | -0.04 | 0.20  *** | -0.06  * | 0.05  * | -0.25  *** | 0.11  *** | 0.69  *** |  |
| 17 | Years in US | -0.07  ** | 0.23  ** | 0.04 | -0.12  ** | 0.02 | 0.33  ** | 0.06  ** | -0.60  ** | 0.29  ** | 0.27  ** | 0.10  ** | -0.03 | 0.06  ** | 0.01 | -0.09  ** | -0.19  *** |

Notes. IADLs = Instrumental activities of daily living; NDI = neighborhood disorder; NCI = neighborhood cohesion; NSI = neighborhood segregation; NSES = neigborhood socioeconomic status;

**Supplemental Table 3.** *LGCM Analysis of Social Relationships, Neighborhood Characteristics, and Global Cognition*

| Predictors | Model 4 | | | | Model 5 | | | | Model 6 | | | |
| --- | --- | --- | --- | --- | --- | --- | --- | --- | --- | --- | --- | --- |
|  | Intercept | | Slope | | Intercept | | Slope | | Intercept | | Slope | |
|  | Estimate | SE | Estimate | SE | Estimate | SE | Estimate | SE | Estimate | SE | Estimate | SE |
| Disconnected | -0.019 | 0.019 | 0.002 | 0.004 | -0.017 | 0.012 | 0.002 | 0.004 | -0.023 | 0.012 | 0.001 | 0.004 |
| Loneliness | 0.030* | 0.012 | -0.007** | 0.002 | 0.030** | 0.009 | -0.007** | 0.002 | 0.030*** | 0.009 | -0.007** | 0.002 |
| Social engagement | 0.016*** | 0.002 | 0.000 | 0.000 | 0.041*** | 0.002 | 0.000 | 0.000 | 0.017*** | 0.002 | 0.000 | 0.000 |
| Social support | 0.012** | 0.004 | -0.001 | 0.001 | 0.024*** | 0.005 | -0.001 | 0.001 | 0.013*** | 0.003 | -0.001 | 0.001 |
| Age | -0.019*** | 0.002 | -0.003*** | 0.000 | -0.019*** | 0.002 | -0.003*** | 0.000 | -0.018*** | 0.002 | -0.003*** | 0.000 |
| Female | -0.042 | 0.024 | 0.013** | 0.005 | -0.041 | 0.023 | 0.013** | 0.004 | -0.037 | 0.023 | 0.013*** | 0.004 |
| Income | 0.029* | 0.011 | -0.001 | 0.002 | 0.029** | 0.011 | -0.001 | 0.002 | 0.028** | 0.011 | -0.001 | 0.002 |
| Education | 0.061*** | 0.003 | 0.002*** | 0.001 | 0.060*** | 0.003 | 0.002*** | 0.001 | 0.061*** | 0.003 | 0.002*** | 0.001 |
| Acculturation | 0.006* | 0.004 | 0.001 | 0.001 | 0.006 | 0.004 | 0.001 | 0.000 | 0.007* | 0.004 | 0.001 | 0.001 |
| Years in US | 0.002 | 0.001 | 0.000 | 0.000 | 0.002* | 0.001 | 0.000 | 0.000 | 0.002 | 0.001 | 0.000 | 0.000 |
| IADLs | -0.032*** | 0.003 | -0.001 | 0.001 | -0.032*** | 0.003 | -0.001 | 0.001 | -0.032*** | 0.003 | -0.001 | 0.001 |
| Depression | -0.006 | 0.004 | 0.001 | 0.001 | -0.006 | 0.003 | 0.001 | 0.001 | -0.007* | 0.003 | 0.001 | 0.001 |
| **NCI** | 0.011 | 0.015 | -0.003 | 0.003 |  |  |  |  |  |  |  |  |
| **NSI** |  |  |  |  | 0.025 | 0.033 | 0.002 | 0.004 |  |  |  |  |
| **NDI** |  |  |  |  |  |  |  |  | 0.005* | 0.002 | 0.000 | 0.001 |
| **Model fit** |  |  |  |  |  |  |  |  |  |  |  |  |
| χ²(df) | 188.56*** (34) | | | | 170.91*** (34) | | | | 175.60*** (34) | | | |
| CFI | 0.978 | | | | 0.993 | | | | 0.993 | | | |
| TLI | 0.962 | | | | 0.988 | | | | 0.988 | | | |
| RMSEA | 0.047 | | | | 0.044 | | | | 0.045 | | | |

*Note.* IADLs = Instrumental activities of daily living; NDI = neighborhood disorder; NCI = neighborhood cohesion; NSI = neighborhood segregation; NSES = neigborhood socioeconomic status; TLI = Tucker–Lewis index; CFI = comparative fit index; RMSEA = root mean square error of approximation. Model estimate and standard error were reported.

**p <* .05. ***p <* .01. ****p <* .001.

**Supplementary Table 4.** *Latent Growth Curve Modeling* *Models of Social Relationships, Neighborhood Contexts, and Global Cognition Controlling for Time-varying Covariates*

| Predictors | Model 2 | | | | Model 3-6 | | | |
| --- | --- | --- | --- | --- | --- | --- | --- | --- |
|  | Intercept | | Slope | | Intercept | | Slope | |
|  | Estimate | SE | Estimate | SE | Estimate | SE | Estimate | SE |
| Disconnectedness | -0.187*** | 0.022 | -0.005 | 0.004 | -0.034** | 0.010 | 0.002 | 0.003 |
| Loneliness | 0.022# | 0.013 | -0.005* | 0.002 | 0.014 | 0.010 | -0.007*** | 0.002 |
| Social engagement T1 on cognition T1 | 0.020*** | 0.001 |  |  | 0.011*** | 0.001 |  |  |
| Social engagement T2 on cognition T2 | 0.018*** | 0.001 |  |  | 0.011*** | 0.002 |  |  |
| Social engagement T3 on cognition T3 | 0.028*** | 0.002 |  |  | 0.019*** | 0.002 |  |  |
| Social engagement T4 on cognition T4 | 0.031*** | 0.002 |  |  | 0.023*** | 0.002 |  |  |
| Social support T1 on cognition T1 | 0.004 | 0.003 |  |  | 0.000 | 0.004 |  |  |
| Social support T2 on cognition T2 | 0.015*** | 0.003 |  |  | 0.007 | 0.005 |  |  |
| Social support T3 on cognition T3 | 0.010** | 0.003 |  |  | -0.003 | 0.003 |  |  |
| Social support T4 on cognition T4 | 0.007* | 0.002 |  |  | -0.003 | 0.003 |  |  |
| Age |  |  |  |  | -0.022*** | 0.001 | -0.002*** | 0.000 |
| Female |  |  |  |  | -0.058** | 0.019 | 0.008* | 0.003 |
| Income |  |  |  |  | 0.029* | 0.012 | -0.002 | 0.002 |
| Education |  |  |  |  | 0.070*** | 0.003 | 0.001 | 0.001 |
| Acculturation |  |  |  |  | 0.009** | 0.004 | 0.000 | 0.001 |
| Years in US |  |  |  |  | 0.002* | 0.001 | 0.000 | 0.000 |
| IADL T1 on cognition T1 |  |  |  |  | -0.019*** | 0.003 |  |  |
| IADL T2 on cognition T2 |  |  |  |  | -0.020*** | 0.003 |  |  |
| IADL T3 on cognition T3 |  |  |  |  | -0.011*** | 0.002 |  |  |
| IADL T4 on cognition T4 |  |  |  |  | -0.022*** | 0.002 |  |  |
| Depression T1 on cognition T1 |  |  |  |  | -0.012** | 0.004 |  |  |
| Depression T2 on cognition T2 |  |  |  |  | -0.005 | 0.003 |  |  |
| Depression T3 on cognition T3 |  |  |  |  | -0.013*** | 0.003 |  |  |
| Depression T4 on cognition T4 |  |  |  |  | -0.007* | 0.003 |  |  |
| NSES |  |  |  |  | 0.003 | 0.006 | 0.001 | 0.001 |
| NCI^4^ |  |  |  |  | 0.022# | 0.012 | -0.001 | 0.003 |
| NSI^5^ |  |  |  |  | 0.046 | 0.032 | -0.002 | 0.004 |
| NDI^6^ |  |  |  |  | 0.002 | 0.002 | 0.001 | 0.001 |
| **Model fit** |  |  |  |  |  |  |  |  |
| χ²(df) | 724.31(36)*** | | | | 360.59(74)*** | | | |
| CFI | 0.906 | | | | 0.976 | | | |
| TLI | 0.880 | | | | 0.966 | | | |
| RMSEA | 0.097 | | | | 0.045 | | | |

*Note.* IADL = Instrumental activities of daily living; NDI = neighborhood disorder; NCI = neighborhood cohesion; NSI = neighborhood segregation; NSES = neigborhood socioeconomic status; CFI = comparative fit index; TLI = Tucker–Lewis index; RMSEA = root mean square error of approximation. Model estimate and standard error were reported. Model 3 included NSES and individual-level covariates. NDI, NCI, and NSI were estimated in separate Models 4-6 with individual-level variables. Sample sizes ranged from 1,893 to 2,050 in Models 2-6.

# *p* < .10. **p <* .05. ***p <* .01. ****p <* .001.

**Supplementary Table 5.** *Moderation Effects of Social Relationships and Neighborhood Characteristics on Global Cognition*

| Interactive Terms | Intercept | Slope |
| --- | --- | --- |
|  | Estimate (SE) | Estimate (SE) |
| Disconnect*NCI | -0.011(0.025) | 0.006(0.004) |
| Loneliness*NCI | 0.010(0.019) | 0.002(0.003) |
| Disconnect*NDI | -0.002(0.003) | 0.002(0.001)* |
| Loneliness*NDI | 0.006(0.002)** | -0.001(.000)* |
| Disconnect*NSES | 0.001(0.004) | -0.002(0.000)# |
| Loneliness*NSES | -0.001(0.003) | 0.001(0.001)# |
| Disconnect*NSI | -0.019(0.020) | -0.016(0.004)*** |
| Loneliness*NSI | 0.011(0.013) | 0.004(0.003) |

*Note.* NDI = neighborhood disorder; NCI = neighborhood cohesion; NSI = neighborhood segregation; NSES = neigborhood socioeconomic status. Eight separate models were estimated with each interaction term after controlling for time-invariant and time-varying covariates.

*#p<*.10*, *p <* .05. ***p <* .01. ****p <* .001.
